# BRASH syndrome in a patient with coexisting anxiety disorder and long-term benzodiazepine use: A case report

**DOI:** 10.21542/gcsp.2025.40

**Published:** 2025-08-30

**Authors:** Kellyn Trycia Zenjaya, I Gede Putu Bayu Tirta Permana, Ragil Nur Rosyadi

**Affiliations:** 1 Faculty of Medicine, Hang Tuah University, Surabaya, Indonesia; 2 Department of Cardiology and Vascular Medicine, Dr. Ramelan Naval Centre Hospital, Faculty of Medicine, Hang Tuah University, Surabaya, Indonesia

## Abstract

Background:  BRASH syndrome represents a constellation of symptoms characterized by bradycardia, renal impairment, AV block, shock, and hyperkalemia. This vicious cycle leads to rapid clinical worsening and requires optimal management. This case illustrates the importance of rapid decision-making and management to navigate the critical phase of BRASH syndrome.

Case Summary: A 73-year-old woman with a pre-existing anxiety disorder presented with sudden generalized fatigue and several episodes of dyspnea. The initial electrocardiogram (ECG) revealed bradycardia (30 beats/min) with episodes of sinus arrest, RBBB, and PVC bigeminy. Laboratory tests indicated hyperkalemia, hyponatremia, elevated blood urea nitrogen, and elevated creatinine levels. Long-term alprazolam and recent bisoprolol use were documented in this patient. Dopamine was administered for inotropic and vasopressor support. Calcium gluconate was given, and a temporary pacemaker was considered. After three days of hospitalization, the patient’s condition improved, with an ECG showing a heart rate of 66 beats per minute and improved renal function following the correction of hyperkalemia.

Discussion: We recommend considering BRASH syndrome in patients presenting with bradycardia and hyperkalemia with recent AV nodal blocking agent use. In our case, bisoprolol was the most likely precipitating factor. The patient’s long-term alprazolam use was also noted; while any potential modulatory role remains speculative, it may warrant further investigation. It is important to recognize this so that appropriate synergistic treatment for all contributing factors can be implemented, rather than focusing on a single component.

## Background

Cardiovascular disease (CVD) is a substantial threat to middle-aged and older individuals, with a profound impact on morbidity and mortality, as evidenced by the staggering toll of 18.6 million deaths recorded in 2019^1^. Patients with CVD present with chest discomfort, palpitations, bradycardia, and shortness of breath^2^.

BRASH syndrome represents a constellation of symptoms characterized by bradycardia, renal impairment, AV block, shock, and hyperkalemia^3^. Profound bradycardia is observed in patients taking AV nodal blockers who present with acute kidney injury and hyperkalemia^4^. The synergistic combination of AV block and hyperkalemia describes the role of pathophysiology in BRASH syndrome^5^. This combination creates a cycle leading to rapid clinical worsening^6^. Lack of knowledge about this cycle leads to suboptimal management, and upon discharge, reinstating prior medications can lead to recurrent hospitalizations and unnecessary treatments^4^.

Patients with BRASH syndrome present with various clinical presentations leading to misdiagnosis and treatment without understanding the underlying mechanism^6,7,8^. The prevalence of this syndrome is not fully described. The increasing aging population alongside proactive healthcare preventive measures such as rigorous blood pressure control necessitates a deeper understanding of this syndrome’s symptoms and optimal treatment strategies^4^.

We present a case of a 73-year-old female with BRASH syndrome with several episodes of sinus arrest. This occurred alongside a pre-existing anxiety disorder, prompting an investigation into the potential connection between anxiety disorder and BRASH syndrome. To our knowledge, this is the first reported case of BRASH syndrome occurring in a patient with anxiety disorder.

## Case presentation

A 73-year-old woman presented to the emergency department with sudden generalized fatigue and trembling for 12 h before being admitted to the hospital. The family stated that the patient was discovered in bed, appearing pale and with cold extremities. One day prior to her arrival, she experienced heart palpitations and multiple episodes of shortness of breath with poor oral intake. She denied any loss of consciousness, fever, chest pain, or body swelling. The patient was known to have a past medical history of type 2 diabetes mellitus for 20 years and congestive heart failure (CHF) New York Heart Association (NYHA) stage 3 for the last 2 years. The patient denied any kidney failure. She was compliant with her home medications, which included nifedipine 30 mg once daily, amlodipine 10 mg once daily, candesartan 16 mg once daily for her CHF, metformin 500 mg three times a day, glimepiride 2 mg once daily, and premixed insulin (Ryzodeg) 10 U twice daily for her type 2 diabetes mellitus. She restricted her water intake. The patient had also been referred to a psychiatrist due to her insomnia and her agitation to loud noises since one year ago. She was diagnosed with an unspecified anxiety disorder and has since taken alprazolam 1 mg nightly on a routine basis. No other pharmacologic treatments such as SSRIs or non-pharmacologic interventions (e.g., cognitive behavioral therapy) were attempted. She was given the medication bisoprolol 2.5 mg and has been taking it for the previous month after experiencing breathing difficulties and leg edema one month before her admission. She had been prescribed bisoprolol around five months prior and had stopped taking it after one month on the doctor’s recommendation.

**Figure 1.**
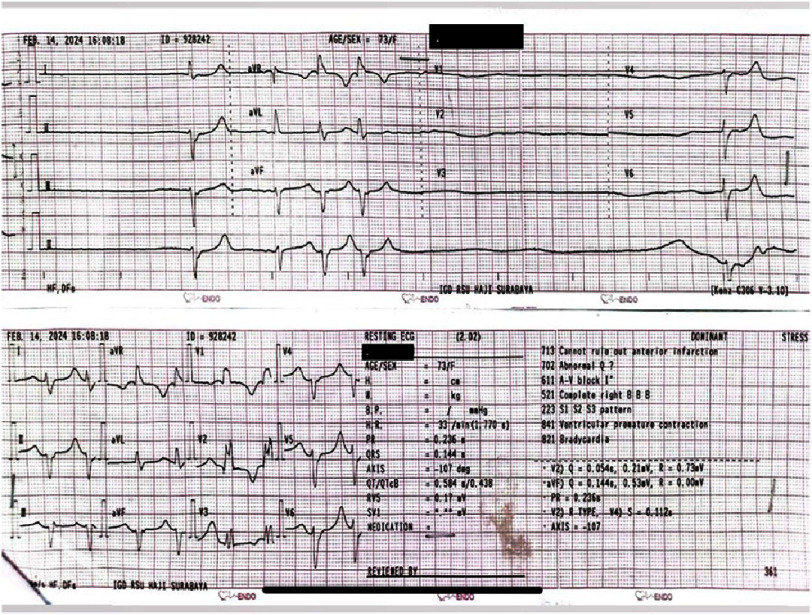
Patient’s electrocardiogram in the emergency department.

On evaluation, the patient was lethargic with a blood pressure of 122/61 mmHg, a temperature of 36.3 °C, a weak pulse with a heart rate of 39 beats per minute, and cold extremities. The electrocardiogram (ECG) was remarkable for bradycardia (30 beats/min) with episodes of sinus arrest, right bundle branch block (RBBB), and bigeminal premature ventricular contractions (PVCs). The ECG can be seen in Figure 1. The patient’s laboratory results were notable for hyperkalemia of 7.3 mmol/L (normal range: 3.6–5.0 mmol/L), elevated blood urea nitrogen (BUN) of 90 mg/dL (normal range: 6–20 mg/dL), elevated serum creatinine of 3.1 mg/dL (normal range: 0.6–1.2 mg/dL), low sodium level of 129 mmol/L (normal range: 136–145 mmol/L), and elevated leukocyte count of 16,220/mm^3^ (normal range: 4,500–11,000/mm^3^). She had a normal blood glucose level. A chest X-ray revealed cardiomegaly and an increased bronchovascular pattern. Blood gas analysis showed pH 7.465 (normal range 7.35–7.45), low PCO_2_ of 29.5 mmHg (normal range 35–45 mmHg), base excess of −3 mmol/L (normal range −2 to +2), and normal HCO_3_ of 22.6 mEq/L. The patient was then diagnosed with sick sinus syndrome caused by an electrolyte imbalance.

At the emergency department, a dopamine drip of 5 mcg/min was initiated to provide inotropic and vasopressor support, as her bradycardia was not responsive to atropine. The patient was placed on 3 L/min of O_2_ through a nasal cannula. Additionally, 10 U of rapid-acting insulin (NovoRapid) with 500 mL of dextrose (10%), 14 drops/min, and calcium (Ca) gluconate (3 ampoules) were administered. The patient was then admitted to the Intensive Cardiac Care Unit (ICCU) for further evaluation and treatment, with consideration for temporary pacemaker (TPM) placement. After 2 days of administration, the clinical condition continued to improve, and the patient was successfully weaned off the dopamine infusion. Calcium gluconate infusion was stopped after 4 days of administration. Due to the improvement of her bradycardia and several other considerations, TPM was not installed in the patient. Laboratory values from admission time and after ICCU admission can be seen in Table 1. The patient’s condition has remarkably improved and is stable. ECG after 3 days of admission shows a heart rate of 66 beats per minute. As the hyperkalemia was corrected, the patient’s renal function also improved. BUN and serum creatinine declined. The patient was discharged after 5 days of hospital admission and advised to avoid further use of any beta-blockers, continue the rest of her home medications, and routinely follow up with the Cardiology Department. At the one-week outpatient follow-up, she remained asymptomatic with a resting sinus rhythm of 58 beats per minute. No further episodes of bradycardia, dizziness, or hypotension were reported.

**Table 1:** Laboratory values from admission time and 3 days after admission with references.

Lab results	At admission	3 days after admission	Reference
Kalium (mmol/L)	7.3	4.29	3.6–5
Natrium (mmol/L)	129	136.9	136–145
BUN (mg/dl)	90	27	6-20
Creatinin (mg/dl)	3.1	0.9	0.6–1.2

## Discussion

This report presents a case of BRASH syndrome in a patient with anxiety disorder. BRASH syndrome is characterized by a clinical presentation of bradycardia, renal failure, atrioventricular (AV)-nodal blockade, shock, and hyperkalemia^3,9,10^. This condition is typically due to the synergistic mechanism between hyperkalemia and AV-nodal blocking medications, which therefore leads to bradycardia. Bradycardia directly reduces cardiac output, which may decrease renal perfusion and may cause kidney injury. Kidney injury exacerbates hyperkalemia due to impaired potassium secretion by the kidney^10^. The hyperkalemic state is proposed to synergistically enhance the effect of AV-blocking medication within the body to exacerbate bradycardia^5^. The symptomatology for BRASH syndrome varies around symptomatic bradycardia, generalized weakness, dyspnea, dizziness, syncope, altered mental status, and others, depending on how far this cycle progresses^11^. The pathophysiology underlying BRASH syndrome has been known since the 1990s, but was specifically recognized as a new entity in 2016 by Joshua Farkas^10,12^. Left unrecognized and uncontrolled, this vicious cycle may lead to multiorgan failure due to the progressiveness of hypoperfusion^9,10^. Usually, significant increases in potassium levels (>7) are necessary for isolated hyperkalemia to result in bradycardia^9^.

However, patients with BRASH syndrome may have mild hyperkalemia yet be adherent to AV-nodal blocking agents. ECG for BRASH syndrome usually demonstrates bradycardia without the classic features shown in hyperkalemia (e.g., peaked T waves^3,10^). Therefore, these clues are important to differentiate between isolated hyperkalemia and BRASH syndrome. In our case, the patient was recently prescribed bisoprolol and took it routinely for one month prior to hospital admission. The patient denied experiencing any symptoms like this before. We believe that the patient developed BRASH syndrome, triggered by the bisoprolol that she had taken recently. This condition may be aggravated by CHF and its medications. Bradycardia can be induced by the intoxication of a beta-blocking agent (BB) or a calcium channel blocker (CCB). To rule this out, it is important to review the patient’s clinical history. Patients with BRASH syndrome commonly take their medication at a certain dose as directed. Both acute kidney injury (AKI) and chronic kidney disease (CKD) may induce BRASH syndrome, but they may have different mechanisms. Pre-renal AKI likely developed as a result of BRASH syndrome, while CKD can exacerbate and hasten the progression of BRASH syndrome. Based on the BUN-to-creatinine ratio (>20), the patient is suspected of having a pre-renal acute kidney injury. Renal function tests in our patient likewise significantly improved after treatment. However, further examination of the kidney may be necessary.

In our case, the ECG shows that the bradycardia contains several episodes of sinus arrest, followed by some premature ventricular contraction (PVC) rhythm. This condition is referred to as sick sinus syndrome (SSS), which defines any sinus pause or arrest of 3 s or more without atrial activity^13^. The impairment of atrial activity can come from various mechanisms, including cardiac electrical dysfunction, cardiac structural abnormalities, autonomic dysfunction, and metabolic imbalance^13^. Therefore, it is necessary to rule out any abnormality contributing to sick sinus syndrome. The initial impression of sick sinus syndrome was revised after identifying key features of BRASH syndrome, including hyperkalemia, renal dysfunction, hypotension, and recent AV-nodal blocker use, all of which resolved with appropriate medical management without requiring pacemaker implantation. Renal function tests and serum electrolytes are highly valuable, especially in patients consuming AV blocking agents. The patient in this case had an anxiety disorder and was routinely taking alprazolam 1 mg daily. The bradycardia in our case was most likely attributable to AV-nodal blocking medication. Interestingly, this patient has been on bisoprolol therapy, a beta-blocker that may play a central role in the development of BRASH syndrome^14^. Beta-blockers are well recognized for their ability to slow AV nodal conduction by blocking beta-adrenergic receptors, resulting in bradycardia. When this slowing of the heart rate reduces renal perfusion, it creates a cycle of worsening kidney injury and hyperkalemia^15^. The negative inotropic and chronotropic effects of beta-blockers can lower effective arterial blood volume, further decreasing renal perfusion and contributing to acute-on-chronic kidney failure. This reduced kidney function impairs the elimination of both beta-blockers and potassium, thereby perpetuating the cycle of bradycardia and hyperkalemia^16^. Although BRASH syndrome is often attributed to the accumulation of AV-nodal blocking agents in the setting of renal dysfunction, recent literature has questioned this mechanism. A 2024 case report in JEM Reports noted that serum concentrations of metoprolol remained far below toxic thresholds, despite classic BRASH features. The authors emphasized that the current BRASH model may overgeneralize the role of AV-nodal blockers, particularly in the absence of direct pharmacokinetic evidence^18^. In addition to the chronic use of beta-blockers and alprazolam, this patient also exhibited poor oral intake and clinical signs of fluid retention secondary to heart failure. Hypovolemia is a common factor that complicates the pathophysiology of BRASH syndrome, especially in elderly individuals^10^. Hypovolemia may arise from reduced fluid consumption, diuretic therapy, or other causes of fluid loss. When intravascular volume drops, renal perfusion declines, impairing the kidney’s capacity to eliminate potassium and enhancing the effects of AV nodal blocking agents^17^.

**Figure 2.**
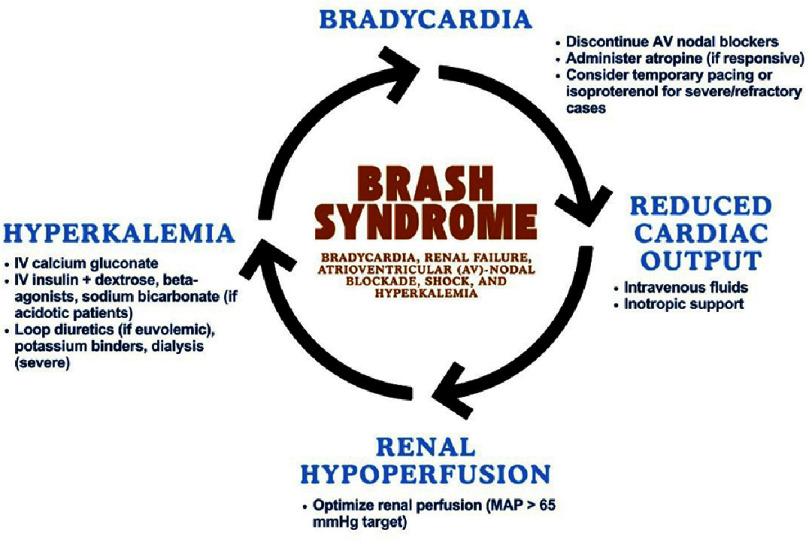
Pathophysiological cycle of BRASH syndrome and key intervention points.

Hemodynamic support is essential to prevent multiorgan failure caused by the progression of the BRASH cycle. Hyperkalemia remains the key to this mechanism that causes bradycardia. To prevent tissue hypoperfusion, particularly in the kidney, the treatment for bradycardia itself is equally crucial. BRASH syndrome requires a multipronged treatment approach targeting all components simultaneously. Management includes fluid resuscitation and vasopressors for hemodynamic support, insulin-dextrose and calcium gluconate for hyperkalemia, and discontinuation of AV nodal blockers. Mild cases often respond to fluids and calcium, while moderate cases may require repeated calcium doses, sodium bicarbonate, or beta-2 agonists. Most patients improve with these general measures, but refractory cases may need advanced therapies^3,5,9,16^. In this instance, if the vicious cycle is not managed, it could deteriorate, necessitating intensive treatment such as TPM and hemodialysis^10^. The vicious cycle of BRASH syndrome and each intervention point are illustrated in Figure 2.

## Limitations

This report represents a single-patient observation of a BRASH syndrome patient with long-term use of alprazolam. The association between alprazolam use and bradycardia in this case remains speculative. While temporally related, causality cannot be inferred due to the lack of mechanistic evidence and potential confounding factors such as autonomic dysregulation related to anxiety. Prior reports^19–21^ describe similar observations, but are limited to isolated case studies without systematic evaluation. Given the anecdotal nature of the existing literature, further investigation is required to clarify any potential causal relationship between benzodiazepine use and cardiac conduction abnormalities.

### What have we learned?

This case highlights the relationship between hyperkalemia, AV-nodal blocking medications, and bradycardia in the onset of BRASH syndrome in a patient with an anxiety disorder and prolonged alprazolam use. The administration of bisoprolol, which exacerbated her condition, underscores the critical importance of early identification of BRASH syndrome to prevent progression to multiorgan failure, emphasizing the necessity for a multidisciplinary approach for effective management and prevention of recurrence.
